# Peritoneal metastases from colorectal cancer belong to Consensus Molecular Subtype 4 and are sensitised to oxaliplatin by inhibiting reducing capacity

**DOI:** 10.1038/s41416-022-01742-5

**Published:** 2022-02-22

**Authors:** Jamila Laoukili, Alexander Constantinides, Emma C. E. Wassenaar, Sjoerd G. Elias, Danielle A. E. Raats, Susanne J. van Schelven, Jonathan van Wettum, Richard Volckmann, Jan Koster, Alwin D. R. Huitema, Simon W. Nienhuijs, Ignace H. J. T. de Hingh, René J. Wiezer, Helma M. U. van Grevenstein, Inne H. M. Borel Rinkes, Djamila Boerma, Onno Kranenburg

**Affiliations:** 1 Department of Surgical Oncology, University Medical Center Utrecht, Utrecht, the Netherlands; 2 Department of Surgery, St. Antonius Hospital, Nieuwegein, The Netherlands; 3 Department of Epidemiology, Julius Center, University Medical Center Utrecht, Utrecht, the Netherlands; 4 Utrecht Platform for Organoid Technology, Utrecht University, Utrecht, the Netherlands; 5 Department of Oncogenomics, Amsterdam UMC, Amsterdam, The Netherlands; 6 Department of Pharmacy and Pharmacology, Antoni van Leeuwenhoek Hospital, Amsterdam, The Netherlands; 7 Department of Clinical Pharmacy, University Medical Centre, Utrecht, the Netherlands; 8 Department of Pharmacology, Princess Máxima Center for Pediatric Oncology, Utrecht, The Netherlands; 9 Department of Surgery, Catharina Hospital, Eindhoven, The Netherlands; 10 School for Oncology and Developmental Biology, GROW, Maastricht, The Netherlands

**Keywords:** Cancer therapeutic resistance, Colorectal cancer

## Abstract

**Background:**

Peritoneal metastases (PM) in colorectal cancer (CRC) are associated with therapy resistance and poor survival. Oxaliplatin monotherapy is widely applied in the intraperitoneal treatment of PM, but fails to yield clinical benefit. We aimed to identify the mechanism(s) underlying PM resistance to oxaliplatin and to develop strategies overcoming such resistance.

**Experimental design:**

We generated a biobank consisting of 35 primary tumour regions and 59 paired PM from 12 patients. All samples were analysed by RNA sequencing. We also generated a series of PM-derived organoid (PMDO) cultures and used these to design and test strategies to overcome resistance to oxaliplatin.

**Results:**

PM displayed various hallmarks of aggressive CRC biology. The vast majority of PM and paired primary tumours belonged to the Consensus Molecular Subtype 4 (CMS4). PMDO cultures were resistant to oxaliplatin and expressed high levels of glutamate-cysteine ligase (GCLC) causing detoxification of oxaliplatin through glutathione synthesis. Genetic or pharmacological targeting of GCLC sensitised PMDOs to a 1-h exposure to oxaliplatin, through increased platinum-DNA adduct formation.

**Conclusions:**

These results link oxaliplatin resistance of colorectal PM to their CMS4 status and high reducing capacity. Inhibiting the reducing capacity of PM may be an effective strategy to overcome PM resistance to oxaliplatin.

## Graphical abstract

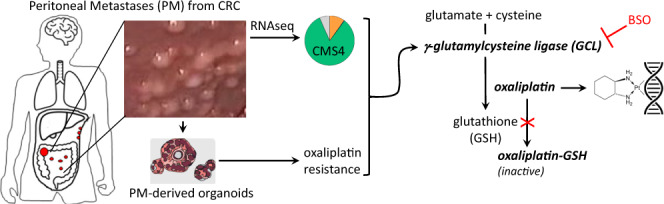

## Introduction

Systemic chemotherapy of metastatic colorectal cancer (mCRC) has prolonged median overall survival to over 2 years. However, patients with metastases in the peritoneal cavity (peritoneal metastases, PM) have a significantly reduced benefit from systemic chemotherapy compared with metastases at other sites [1, 2]. The mechanisms underlying this site-dependent variation in treatment benefit are unknown and may be related to differences in tumour biology and/or drug exposure. Of particular interest is the molecular classification of CRC, based on RNA expression patterns, yielding four consensus molecular subtypes (CMS1-4) [3]. We have previously developed a novel 4-gene RTqPCR test to identify CMS4 tumours [4]. By applying this test to a series of PM and paired primary tumours, we demonstrated an enrichment of CMS4 in this subgroup of CRC patients [5]. In general, CMS4 CRC is characterised by a mesenchymal phenotype, poor prognosis, and a poor response to systemic therapies [3, 6–9].

The enclosed nature of the peritoneal cavity provides a unique opportunity to treat PM locally, by intraperitoneal chemotherapy. This is now routinely applied as an adjuvant treatment in patients with operable PM by hyperthermic intraperitoneal chemotherapy (HIPEC), aiming to kill residual local micro-metastases. However, a recent clinical study failed to show the additional benefit of adjuvant HIPEC with oxaliplatin monotherapy over surgery alone [10]. Intraperitoneal chemotherapy with oxaliplatin is currently also being evaluated in patients with inoperable PM. The most commonly applied procedure in this patient category is pressurised intraperitoneal aerosolized chemotherapy (PIPAC). During PIPAC, oxaliplatin is applied as an aerosol to achieve homogenous distribution throughout the peritoneal cavity. The procedure has proven to be safe and well-tolerated [11], but clinical benefit has yet to be demonstrated.

Importantly, a biological rationale for the use of oxaliplatin as a single drug in the intraperitoneal treatment of PM (HIPEC or PIPAC) is lacking. We previously demonstrated that PM-derived organoids (PMDOs) from cancer patients are resistant to clinically relevant doses of oxaliplatin [12]. We hypothesised that this may be due to an intrinsic resistance to chemotherapy, related to the predominant CMS4 status of PM [5]. The diagnostic test that we have previously applied to demonstrate an enrichment of CMS4 in patients with PM measures expression of only 4 genes [4, 5]. Although this test is a robust diagnostic tool that can be easily implemented in most molecular pathology labs, it was not designed to yield detailed biological information relating—for instance—to drug resistance.

Therefore, the aims of this study were to gain a detailed insight into the distinguishing features of PM, in order to identify potential mechanisms of resistance to oxaliplatin. In addition, we applied organoid technology [13–15] to generate an in vitro platform for designing and testing therapeutic strategies that may overcome such resistance. Organoid technology is currently the only pre-clinical model system in which therapy responses observed in cancer patients are faithfully reproduced in vitro [16–19].

## Materials and methods

### Biobank

In the St. Antonius Hospital, a tertiary referral centre for the surgical treatment of peritoneal metastasis of colorectal cancer, a prospective database was collected with patients suitable for inclusion in the HIPEC biobank. The HIPEC biobank (protocol not published but approved by the Medical Research Ethics Committees United (MEC-U, Nieuwegein, the Netherlands) contains patients with paired samples from 13 patients undergoing cytoreductive surgery and hyperthermic intraperitoneal chemotherapy (CRS+HIPEC) for peritoneal metastazied colorectal cancer.

Samples were collected during laparotomy and immediately transferred to the pathology department. In the pathology department, the samples were freshly dissected and snap-frozen in liquid nitrogen before storing at −80 °C. The biobanking process was highly standardised with a dedicated researcher (EW) being present during all operations, transporting the resection specimens to the pathology department for tissue sampling as quickly as possible following resection, in order to keep warm ischaemia times to an absolute minimum. Although variation of this parameter between cases was inevitable, it was not separately registered. However, all samples were processed and snap-frozen within one hour after resection. The primary tumour was dissected preferably into three different regions. Metastatic tumour tissue was macroscopically stripped from normal tissue prior to snap freezing.

### RNA sequencing and CMS calling

Frozen tissue samples were cut in 20–30-μm thick cryosections with a cryostat and immersed in RLT buffer (RNeasy^®^ Mini Kit; Qiagen, Stockholm, Sweden) plus 1 per cent β-mercaptoethanol. RNA isolation, including on-column DNase digestion, was performed according to the manufacturer’s instructions. RNA concentration was measured using a NanoDrop™ 2000 instrument (Thermo Fisher Scientific). Samples with RNA integrity (RIN) values below 8 were excluded from further analysis, resulting in the analysis of 59 PM samples and 35 primary tumour regions from 12 individual patients.

Generation of sequencing libraries was performed using the Truseq RNA stranded poly A Library Preparation Kit (Illumina, San Diego, CA, USA). Sequencing was performed on Illumina NextSeq500 with 75-bp reads (Illumina). The average sequencing depth (total number of reads) was 28.3 M reads (range 25.1–36.3 M).

Samples were classified into molecular subtypes using the Random Forest (RF) method available in the R package of the CMS classifier (v1·0·0, https://www.synapse.org/#!Synapse:syn4961785).

In order to normalise the data of these 12 patients, we used a ‘piggyback cohort’ of 196 primary tumour samples of colorectal cancer with varying disease Stage I–IV.

### Bioinformatics analysis

The RNAseq dataset (GSE190609) was uploaded into R2 (http://r2.amc.nl) for subsequent additional bioinformatics analyses. Expression of specific gene sets (e.g. the indicated Hallmark and Immune cell gene sets and the CMS4-identifying random forest (RF) classifier genes) was determined by using the ‘view gene set’ option and storing the resulting meta-gene values. The relate-two-tracks option was used to compare expression of each gene set in the primary tumour-derived and PM-derived sample sets. Genes that were significantly differentially expressed between primary tumours and PM were identified using a cut off of *P* < 0.01 (ANOVA) with multiple testing correction by false discovery rate. The list of genes that was expressed at significantly higher levels in PM (366 genes) was used to generate meta-gene expression values for every sample in the primary tumour-PM dataset (this study), as well as in the original CMS dataset containing 3232 CMS-annotated primary tumours. These values were compared to expression of the CMS4-identfying genes in the random forest classifier in both datasets. The relationship between both sets of meta-gene values was determined by generating scatterplots and accompanying correlation coefficients (*r*) and *P* values. The 366 genes distinguishing PM from primary tumours were also used to cluster the CMS-3232 cohort into two groups (high versus low expression) using the *k*-means method. Survival differences in the generated groups were determined by generating Kaplan–Meier curves and accompanying log-rank *P* values.

### Generation of PM-derived organoids

All PM-derived CRC organoids were generated from ascites fluid, collected in the context of the CRC‐PIPAC trial (NCT03246321) [20] at the Catherina Hospital Eindhoven and at the Sint Antonius Hospital in Nieuwegein, The Netherlands. This study was approved by Medical research Ethics Committees United (MEC-U) in Nieuwegein, The Netherlands. All patients gave their written informed consent after a consideration period.

Intraoperatively ascites was collected, cooled and transported to our culturing lab. After spinning down at 400 × *g* for 5 min to collect the cell pellet, the cells were washed 5 times with PBS, mucus and debris were disposed of, and the single cells were plated out in Matrigel^®^ (Corning, 356231, New York, USA) in droplets. After solidification for 30 min at 37 °C full CRC organoid growth medium was added and refreshed twice a week. Full CRC organoid growth medium consisted of basal medium supplemented with niche factors. The basal medium used was Advanced DMEM/F12 (Gibco, Life Technologies Corporation, 12634-010, Grand Island, NY, USA), supplemented with 400 µM Glutamax (Life Technologies, 35050038), 10 mM HEPES (Lonza, BE17-737E, Basel, Switzerland), and 50 U/mL–50 µg/mL penicillin–streptomycin (5.000 U/mL & 5.000 µg/mL; Life Technologies Corporation, 15070063). The niche factors used were 100 ng/mL noggin conditioned medium, R-Spondin conditioned medium, 1× B27 (Life Technologies, 17504044), 500 nM A83-01 (Biovision, 1725-1, Zurich, Switzerland), 10 µM SB202190 (Sigma-Aldrich, S7067, St Louis, MO, USA), 1,25 mM N-Acetyl-L-cysteine (Sigma-Aldrich, A9165-5G), 10 mM Nicotinamide (Sigma-Aldrich, N0636-100gr), ng/mL EGF (Sigma-Aldrich, E9644), 10 nM Gastrin [Leu15] (Sigma-Aldrich, G9145-0,5 mg), and 10 nM prostaglandin E2 (Tocris, 2296-10, Bio-Techne Ltd., Abingdon, UK).

A total of 60 PIPAC procedures made ascites acquisition possible in 57 procedures. This resulted in the establishment of ten stable PM-derived CRC organoids, collected from six patients. Mycoplasma was tested every 2 months. Newly generated growing organoid cultures were passaged by using mechanical and enzymatic dissociation with TrypLE™ (Gibco, Thermo Scientific, 12604021) and were subsequently stored in liquid nitrogen.

#### Reagents

L-Buthionine-sulfoximine (BSO) was purchased from Santa Cruz Biotechnology (cat#: sc-218630). Reduced GSH and N-Acetyl-L-cysteine (NAC) were purchased from Sigma-Aldrich. Oxaliplatin was provided by Fresenius Kabi (5 mg/mL, Bad Homburg, Germany).

### In Vitro drug screen and cell viability assay

Drug response evaluation was performed on 3–5-day-old organoids depending on the PDO line. PDOs were dissociated into single cells using TrypLE™, washed in PBS, re-suspended in CRC organoid growth medium and plated in BME matrix. Three to five days old organoids were harvested from their matrix using Dispase^®^ (Life technologies, 17105-041) for 15 min at 37 °C. Organoids were subsequently washed with PBS, counted, and re-suspended at a concentration of 30 organoids/µL in either full organoid growth medium or reduced medium (DMEM/F12 (Sigma-Aldrich, D8062), supplemented with 400 µM Glutamax, 10 mM Hepes, 50 U/mL–50 µg/mL penicillin–streptomycin, 1× B27, and 100 ng/mL noggin conditioned medium). 100 µL of organoid suspension was then plated on 96-well plates (Corning, Costar^®^ assay plate, 96-well white with clear bottom, 3903, Kennebunk, ME USA) that were pre-coated with 40 µL Matrigel^®^/BME matrix using the Multidrop™ Combi Reagent Dispenser (Thermo Fisher, 5840300). The plates were left for 3–24 h at 37 °C to let the organoids adhere to the matrix.

For BSO-based screens, 24 h prior to oxaliplatin addition, CRC organoid growth medium was replaced with reduced organoid growth medium. Oxaliplatin was dispensed at a concentration range of 0–900 µM using the Tecan D300e Digital Dispenser (Tecan Trading AG, Switzerland). Conditions and drug concentrations were tested in quadruplicate. For the washout experiments following 1 h treatment with oxalipaltin at 42 °C, PDOs were washed twice with warm PBS and supplemented with full CRC organoid growth medium.

Cell viability was measured 72 h after drug exposure using CellTiter-Glo^®^ 3D Cell Viability Assay (Promega Corporation, G9681, Maddison, WI, USA) according to the manufacturer’s instructions, and luminescence was measured using the SpectraMax^®^ M Series Multi-Mode Microplate Reader (Molecular Devices, LLC).

In vitro drug screen and cell viability assay were performed in triplicates or quadruplicates and repeated at least two times on different days. For the screens, one PM-derived organoid line per patient was selected (*N* = 6) and compared with randomly chosen primary CRC-derived organoids (*N* = 5).

### Re-growth assays

PDO were harvested, dissociated into single cells using TrypLE and counted. In all, 10.000 live single cells were then suspended in 25 µl of 50% v/v solution of Matrigel^®^/BME matrix and plated in a 24-well plate. Twenty-four hours prior to treatment the organoid growth medium was replaced with reduced growth medium, in the absence or presence of BSO. Oxaliplatin was added in the desired concentrations and the plates were incubated for 1 h at 42 °C. Organoids were washed twice with warm PBS and supplemented with fresh full organoid growth medium. Medium was refreshed twice a week and as soon as the control-treated organoids were fully outgrown, organoids were harvested, dissociated into single cells using TrypLE and counted. In total, 10.000 live cells were then replated in 25 µl of 50% v/v solution of Matrigel^®^/BME matrix per 24-well plate. If the cells were too few, all cells were replated. The number of grown colonies were documented after 2–3 weeks of culturing using an inverted microscope (EVOS) and quantification of viable clones was performed using CellTiter-Glo^®^ 3D Cell Viability Assay, and luminescence was measured using SpectraMax M5 microplate reader (Molecular Devices). For the re-growth assay, two oxaliplatin-resistant PM-derived organoids were selected.

### Platinum-DNA adduct formation

Platinium-DNA adduct measurement was performed using inductively coupled plasma-mass spectrometry exactly as previously described [21]. Briefly, organoids were treated with DMSO or with oxaliplatin for 5 h or 24 h in the presence or absence of 2 mM of reduced GSH (Sigma). Organoids were released from the Matrigel^®^/BME matrix by addition of 1 mg/ml dispase II (Invitrogen) for 15–30 min at 37 °C. Organoid pellets were lysed for genomic DNA extraction using DNeasy Blood & Tissue kit from Quiagen according to the manufacturer’s instructions.

### GSH measurement

The equivalent of 3000 organoids were re-suspended in a 100 μl volume of reduced DMEM-F12 growth medium and plated on 40 μl Matrigel^®^/BME matrix pre-coated 96-well plates. Organoids were left for 24 h at 37 °C to let the organoids adhere to the matrix, and subsequently treated with control or with BSO for 24 h. GSH concentration was measured using the GSH-Glo assay (Promega) according to the manufacturer’s instructions, and luminescence was measured using SpectraMax M5 microplate reader (Molecular Devices).

### Western blot analysis

PDOs were harvested at the indicated time points using dispase, washed with PBS and lysed in Laemmli lysis buffer (2.5% SDS, 20% glycerol, 120 mM TRIS pH 6.8). Equal amounts of protein (10–20 μg) were run on SDS-PAA gels transferred onto nitrocellulose membranes (Trans-Blot Turbo, Bio-Rad, Hercules, CA, USA), and incubated with antibodies as described. Primary antibodies for GCLC and β-actin were purchased from Abcam (ab190685) and Novus (NB600-501) respectively. phospho-RPA antibody was purchased from Bethyl Laboratories (A300-245A).

### Data analysis and statistics

Spectramax luminescence data were loaded into GraphPad Prism version 9.0.0 for Windows (GraphPad Software, San Diego, California, USA, www.graphpad.com). The oxaliplatin concentrations were log10-transformed, and luminescence values were normalised and transformed into percentages from the control. The best-fitting IC50 curves were generated using the ‘log(inhibitor) vs. response (three parameters)’ option. All area under the curve (AUC) values were based on actual data points. *T* tests were applied to identify significant differences between the two treatment groups. ANOVA was used to identify a significant difference between more than two groups. Normality was tested with the Shapiro–Wilk test, variance differences were tested with *F* test. If distribution was normal and no difference in variance was observed, the *T* test or ANOVA was applied, otherwise a Mann–Whitney or Kruskal–Wallis was used.

## Results

### A biobank of peritoneal metastases with paired primary tumours and other distant metastases

We prospectively generated a biobank of 94 paired tissue samples derived from patients with operable peritoneal metastases (PM) who were scheduled for cytoreductive surgery. The biobank consists of 35 primary tumour regions and 59 peritoneal metastases from 12 patients (Supplementary Table S1).

### PM express signatures associated with aggressive CRC behaviour

**Fig. 1:**
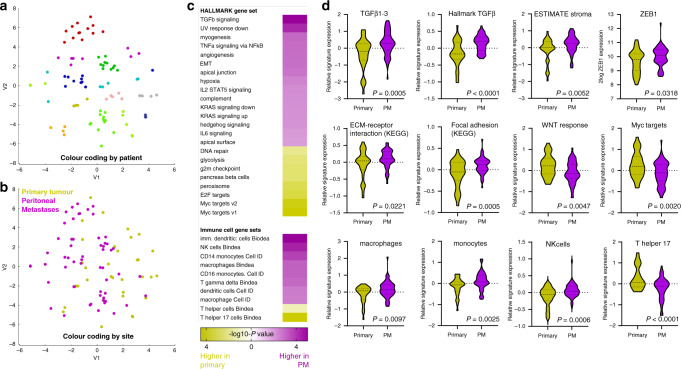
Molecular analysis of colorectal peritoneal metastases reveals multiple features of aggressive tumour behaviour. **a** RNA sequencing of PM (*n* = 59) and paired primary tumour regions (*n* = 35) from 12 individual patients was performed. The t-SNE plot shows the clustering of most samples per patient. **b** The same t-SNE plot colour coded by site (primary versus PM). **c** Gene set enrichment analyses of all Hallmark signatures (*n* = 50) and immune cell gene sets (Bindea and Cell-ID). All gene sets that were significantly enriched in either PM or primary tumour samples are shown and ordered by the significance and direction of enrichment. **d** Examples of box plots of individual gene sets expressed at significantly different levels in either PM or primary tumour samples.

All samples were analysed by RNA sequencing. T-distributed Stochastic Neighbour Embedding (t-SNE) analysis of the resulting dataset revealed that metastases and primary tumours from the same patients mostly cluster together (Fig. 1a). Clustering according to metastasis site or to specific intraperitoneal sub-sites was not observed (Fig. 1b). Patient- and/or tumour-intrinsic (epi-) genetic variables therefore seem to be more important determinants of global gene expression variation than cues from the different tissue microenvironments (colon versus peritoneum).

Next, we analysed differential activation of 50 Hallmark gene signatures representing a series of well-defined biological states [22] in PM versus their paired primary tumours. We identified 15 Hallmark pathways that were expressed at significantly higher levels in PM than in their corresponding primary tumours, including TGF beta signalling, angiogenesis, complement activation and EMT (Fig. 1c, d). This was confirmed by analysis of independent signatures for TGF beta signalling (KEGG) and TGF beta ligands 1-3 (Fig. 1d). High TGF beta signalling in CRC is associated with a fibrotic reaction, characterised by a high content of cancer-associated fibroblasts and lower expression of canonical WNT signalling target genes. Indeed, signatures for stromal fibroblasts (estimate), extracellular matrix remodelling (KEGG) and focal adhesions (KEGG) were significantly higher in PM than in the corresponding primary tumours, while expression of WNT signalling target genes [23] and MYC target genes was significantly lower (Fig. 1d).

Bulk RNA sequencing data can also be used to infer the presence of immune cell types in (colorectal) cancer tissue [24, 25]. We found that signatures reflecting the presence of immature dendritic cells, monocytes, macrophages and NK cells were significantly higher in PM when compared to primary tumours. By contrast, the presence of T helper 17 cells was markedly reduced in PM.

### Peritoneal metastases represent an almost homogeneous entity of CMS4 colorectal cancer

**Fig. 2:**
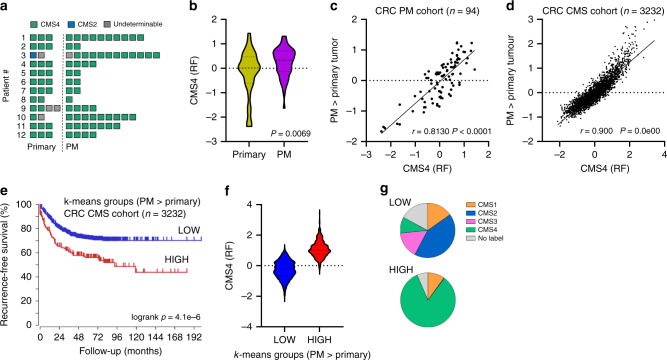
PM and the primary tumours from which they arise represent an almost uniform CMS4 entity within CRC. **a** Application of the CMS classifier on the RNAseq data reveals strong enrichment of CMS4. **b** Expression of the genes positively identifying CMS4 in the original CMS random forest classifier (CMS4 (RF); 143 genes) in PM and primary tumour samples. **c** Differential gene expression analysis identified 366 genes that were significantly higher expressed in PM than in paired primary tumour samples. The scatter plot shows the correlation of meta-gene expression values of this gene set in relation to the CMS4-identifying gene set (CMS4 (RF)). **d** As in (**c**), but in the original composite CMS cohort (*n* = 3232 primary tumours). **e** *k*-means clustering of the CMS cohort using the 366 PM-enriched gene set shows a correlation between high expression of this ‘PM-signature’ and a significantly shorter recurrence-free survival. **f** Expression of CMS4-identifying genes from the RF classifier in the k-means groups generated with the PM signature. **g** Distribution of CMS subtypes in the *k*-means groups generated with the PM signature.

Transcriptome-based classification of CRC has resulted in the identification of 4 consensus molecular subtypes (CMS1-4) [3]. Interestingly, many pathways and cell types that were enriched in PM when compared to primary tumours are also features of CMS4 CRC (e.g.: high TGFβ, angiogenesis, complement, stromal fibroblasts, monocytes, macrophages; low WNT and MYC target genes) [3, 25]. By using an RTqPCR test designed to detect CMS4 [4] we previously demonstrated that the majority of PM (21/28) classified as CMS4 [5]. Application of the original random forest CMS classifier on the new RNAseq dataset showed a near-homogenous classification of all tumours in the PM-CRC cohort as CMS4 (29/34 primary tumour regions, 58/59 peritoneal metastases; Fig. 2a). The sole primary tumour classified as CMS2 gave rise to 12 PM of which 11 were classified as CMS4.

Although virtually all samples analysed were classified as CMS4 we noted that expression of the CMS4-identifying genes from the original random forest classifier (143 genes) varied considerably between groups and was significantly higher in PM than in primary tumours (Fig. 2b), indicating that PM represent a virtually homogeneous CMS4 entity within CRC. We next performed unbiased differential gene expression analysis and identified 366 genes with significantly higher expression in PM when compared to primary tumours (*P* < 0.01), and 138 genes with higher expression in primary tumours (Supplementary Table S2). Expression of the PM > primary gene set was strongly correlated with expression of the 143 CMS4 classifier genes in the CRC-PM cohort analysed in this study (*r* = 0.813), and even better in the large composite CMS cohort consisting of 3232 primary CRC tumours (*r* = 0.900) (Fig. 2c, d). It follows that the major biological difference between PM and primary tumours is a more extreme mesenchymal phenotype in PM. Indeed, when used to cluster a large cohort of primary CRC tumours into low- and high-signature expression groups, the PM > primary gene set identified a poor prognosis subgroup largely consisting of CMS4 tumours (Fig. 2e–g).

Recently, a comparative analysis of 15 PM with 15 paired primary archival tumour samples identified a ‘20-gene peritoneal signature’, of which 15 genes were higher expressed in PM when compared to primary tumours. Expression of this gene set was associated with poor prognosis and, surprisingly, with CMS2 status of the majority of the samples [26]. We found that expression of these PM signature genes correlated extremely well with the 366 genes identified in our study, and with the CMS4 signature genes from the original random forest classifier, both in our CRC-PM cohort and in the original large composite CMS cohort (Supplementary Fig. S1). We conclude that both PM signatures identify CMS4 tumours and that the vast majority of PM belong to CMS4.

We next analysed transcription factor binding sites in the promoter regions of the 366 PM > primary gene set. We found that ZEB1-controlled genes were most significantly enriched in the genes highly expressed in PM (Supplementary Table S3). Moreover, ZEB1 itself was also significantly higher expressed in PM when compared to primary tumours (Fig. 1d). ZEB1 is a master regulator of epithelial-to-mesenchymal transition, and is one of the markers that can be used to identify CMS4 CRC in situ [8].

### PM-derived organoid cultures are characterised by high glutathione synthesis and display resistance to oxaliplatin

**Fig. 3:**
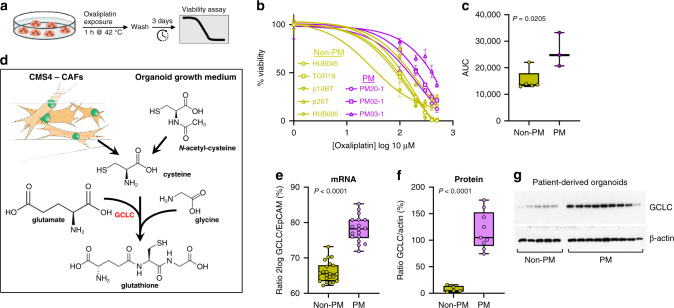
PM-derived organoids display relative resistance to oxaliplatin and express high levels of glutamate-cysteine ligase (GCLC). **a** Schematic representation of the experimental setup. **b** Dose–response curves for five non-PM CRC-derived organoids and threePM-derived organoids. The dose range of oxaliplatin was 1, 100, 200, 300, 400 and 500 μM. Three days after a 1 h exposure to oxaliplatin the fraction of remaining viable cells was measured using Cell Titer Glo and plotted as percentage of untreated control cells. **c** Boxplot showing area under the curve (AUC) values, calculated from the dose–response curves. **d** The contribution of cancer-associated fibroblasts (CAFs) and N-acetyl-cysteine (NAC) in glutathione synthesis. GCLC, the rate-limiting enzyme in this pathway, is indicated in red. **e** GCLC mRNA expression levels in non-PM and PM-derived organoids, measured by RNA sequencing. **f**, **g** GCLC protein expression levels in non-PM and PM-derived organoids in relation to actin. The boxplot in (**f**) shows optical density measurements of the exposed films in (**g**), normalised to actin.

A major challenge in the clinical management of CMS4 CRC is their relative resistance to commonly used drugs in systemic treatment, including oxaliplatin [6–8]. Our finding that CMS4 is overrepresented in PM sheds new light on the recently published results of the PRODIGE7 study, demonstrating a lack of benefit of intraperitoneal oxaliplatin treatment following cytoreductive surgery for PM [10]. To model oxaliplatin resistance of PM, we generated PM-derived organoid (PMDO) cultures from the abdominal fluid of patients who were included in the Dutch PIPAC study, involving intra-abdominal oxaliplatin monotherapy [27]. PMDOs and organoids derived from non-PM CRC tumours were then treated with different concentrations of oxaliplatin for 1 h to generate dose–response curves (Fig. 3a, b). Area under the curve (AUC) analysis revealed that PM-derived organoids were significantly more resistant to oxaliplatin than non-PM-derived organoids (Fig. 3c).

Resistance of stroma-rich tumours (such as CMS4 CRC) to platinum drugs is mediated by the production of cysteine and glutathione by cancer-associated fibroblasts [28] (Fig. 3d). The thiol groups in glutathione coordinate with platinum drugs, causing their direct inactivation and cellular efflux [29]. In organoid cultures, CAFs are lacking as a cysteine source, but the medium is routinely supplemented with N-acetylcysteine (NAC) as an essential precursor for glutathione [14, 30]. The rate-limiting step in glutathione synthesis is the ligation of glutamate with internalised cysteine by glutamate-cysteine ligase (GCLC) (Fig. 3d). Comparative expression analysis of GCLC in PM and non-PM-derived organoids revealed a significantly higher expression of GCLC mRNA and protein in PM-derived organoids (Fig. 3d–f). In line with previous reports [31–33], we found that CMS4 signature genes were primarily expressed by cancer-associated fibroblasts and were largely lost in organoid cultures derived from primary tumours or PM (Supplementary Fig. S2).

### Omission of N-acetylcysteine sensitises PMDOs to oxaliplatin

**Fig. 4:**
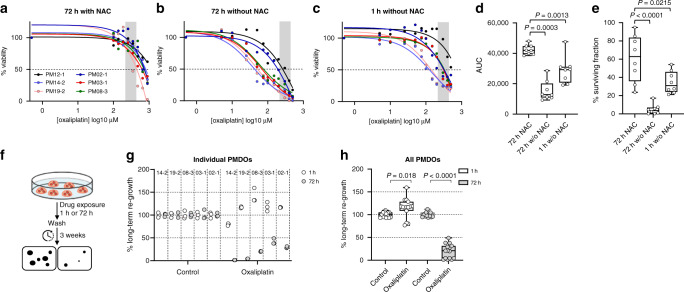
Depletion of NAC sensitises PM-derived organoids to oxaliplatin. **a**–**c** Drug response curves for a series of six distinct PM-derived organoids treated with oxaliplatin for 72 h in the presence (**a**) or in the absence of N-acetyl-cysteine (NAC) (**b**), or treated with oxaliplatin for 1 h in the absence of NAC (**c**). The range of clinically applied oxaliplatin concentrations in the HIPEC perfusate is indicated by the grey area (215–439 μM). **d** Boxplot showing AUC values, calculated from the dose–response curves in (**a**–**c**). **e** Boxplot showing surviving cancer cell fractions calculated from the dose–response curves in (**a**–**c**). **f** Schematic representation of the experimental setup of the long-term regeneration assays. **g** Dotplot showing a quantification of long-term re-growth potential of five distinct PMDOs after a 1-h or 72-h exposure to oxaliplatin (20 μM). **h** Boxplot showing the aggregated data from (**g**).

We next assessed whether glutathione synthesis could modulate PMDO resistance to oxaliplatin. First, we compared standard NAC-supplemented CRC organoid growth medium with NAC-depleted medium in short-term killing assays (Fig. 4a). Omitting NAC from the medium greatly sensitised all PMDOs (*n* = 6) to 72-h exposure to oxaliplatin (Fig. 4a, b). As HIPEC flush times are usually restricted to 30–90 min (rather than 72 h), we next tested the effect of a 1-h exposure of PMDOs to oxaliplatin in the absence of NAC. Strikingly, this short treatment with oxaliplatin in the absence of NAC was significantly more effective in killing PMDOs than a 3-day oxaliplatin treatment in the presence of NAC, as evidenced by significantly lower IC50 values (Fig. 4c), lower AUC values (Fig. 4d), and lower surviving tumour cell fractions (Fig. 4e). In NAC-depleted medium, the IC_50_ values were below the oxaliplatin concentrations used in HIPEC for seven out of eight PMDOs after 3-day drug exposure, and for two out of six PMDOs after a 1-h drug exposure (grey vertical bars in Fig. 4a–c).

The benefit of HIPEC depends on the capacity of drug-exposed tumour cells to regenerate after drug washout. Therefore, we analysed the long-term re-growth potential of oxaliplatin-exposed PMDOs following a 1-h or 3-day exposure to oxaliplatin (Fig. 4f). PMDOs were treated with oxaliplatin in the absence of NAC and were then followed over time, after drug washout. After a 72-h exposure to oxaliplatin three of the five tested PMDOs were able to resume growth within 3 weeks (Fig. 4g). However, after a 1-h treatment, all PMDOs efficiently resumed growth (Fig. 4g). Analysis of re-growth in all PMDOs together revealed a significant reduction of recovery potential in the 72-h-treated group. Interestingly, recovery in the 1-h treated group was even slightly (but significantly) higher than in control-treated PMDOs. We conclude that the omission of NAC significantly sensitises PMDOs to oxaliplatin, but also that a short-term (1-h) treatment is insufficient to generate lasting anti-tumour effects.

### Inhibition of glutathione synthesis allows PMDO eradication by short-term oxaliplatin treatment

**Fig. 5:**
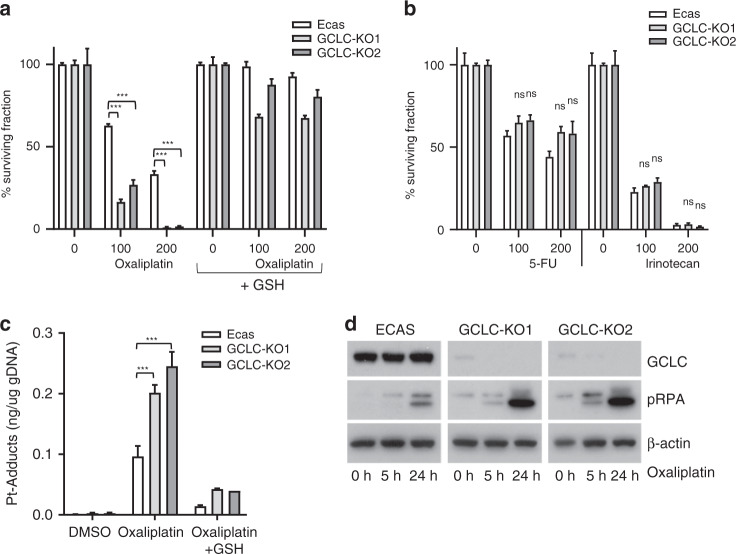
GCLC knockout increases sensitivity to oxaliplatin. **a** Organoids derived from control (ECAS) and two independent GCLC-knockout clones (KO1 and KO2) were seeded in an NAC-depleted growth medium with or without reduced glutathione (GSH, 2 mM) as indicated (*n* = 3 per condition). Cells were treated with oxaliplatin for 72 h at the indicated concentrations (μM), and cell viability was then measured using Titer-Glo assay. The bar graphs show the percentage of surviving cell fractions. All values are plotted as % of untreated controls. **b** As in (**a**), with 5-FU or irinotecan, as indicated. (*n* = 3 per condition). **c** Control and GCLC-knockout organoids were exposed to oxaliplatin (5 h; 200 μM) in the presence or absence of reduced GSH (2 mM). After extensive washing of the cells, genomic DNA was isolated and the platinum (Pt) content was quantified by HPLC. The barplot shows the Pt content (*n* = 3) in ng per ug total genomic DNA. **d** Western blot analysis of pRPA, GCLC and actin protein expression levels in control ECAS and GCLC-knockout organoids following exposure to oxaliplatin (200 μM) at the indicated time points.

We next analysed whether inhibition of glutathione synthesis could further sensitise PMDOs to oxaliplatin. First, we used CMS4-derived organoids (from a patient with PM) in which the catalytic subunit of glutamate-cysteine ligase (GCLC) was deleted using CRISPR-CAS9 technology. Deletion of GCLC sensitised organoids to oxaliplatin in two independently generated knockout clones (Fig. 5a). The addition of exogenous reduced glutathione restored the resistance to oxaliplatin in GCLC-knockout PMDOs (Fig. 5a). We next tested whether GCLC knockout also affected the response of PMDOs to two other drugs frequently used in the systemic treatment of metastatic CRC: 5-fluorouracil (5-FU) and irinotecan. Strikingly, GCLC knockout had no significant effect on the PMDO response to either drug (Fig. 5b) suggesting that glutathione synthesis is an important determinant of PMDO sensitivity to oxaliplatin but not to 5-FU or irinotecan.

Next, we aimed to gain insight into the mechanism of protection of PMDOs against oxaliplatin by GCLC. Direct detoxification of platinum drugs by glutathione could result in reduced DNA adduct formation. To test this hypothesis, we measured the level of platinum-DNA adducts in control and GCLC-knockout PMDOs by a HPLC protocol. We found that GCLC knockout significantly increased Pt-DNA adduct formation, which was blocked by the addition of exogenous glutathione (Fig. 5c). Increased Pt-DNA adduct formation in the GCLC-knockout clones caused an increase in DNA replication stress, as measured by the levels of phosphorylated replication protein A (pRPA) (Fig. 5d).

**Fig. 6:**
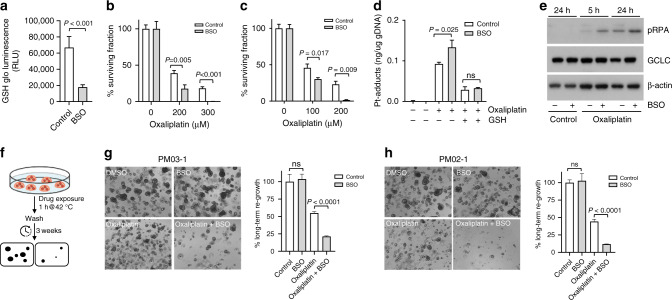
Inhibition of glutathione synthesis sensitises PMDOs to short-term oxaliplatin treatment. **a** PMDOs were treated for 24 h with BSO (100 μM) or solvent. Reduced glutathione (GSH) levels were then measured as described in the 'Materials and methods'. The barplot shows reduced GSH levels in control- and BSO-treated PMDOs. **b**, **c** Two distinct PMDOs were treated for 1 h with oxaliplatin at the indicated concentrations in the presence or absence of BSO (100 μM). The surviving cell fractions were then quantified by Cell Titer Glo. All values are plotted as % of untreated controls. **d** Bar graphs showing a quantification of Pt adducts in the genomic DNA of PMDOs following treatment with oxaliplatin (200 μM) with or without BSO (100 μM) and in the presence or absence of reduced GSH. **e** Western blot analysis of pRPA, GCLC and actin protein expression levels in PMDOs following a 5 or 24 h exposure to oxaliplatin (200 μM) in the presence or absence of BSO. **f** Experimental setup of the long-term regeneration experiments. **g**, **h** Two distinct PDOs were treated with oxaliplatin (200 μM) and BSO (100 μM) alone and in combination for 1 h at 42 °C. Three weeks after drug washout the regenerative capacity was assessed in all conditions by Cell Titer Glo measurements and photographs.

To block glutathione synthesis pharmacologically we next used buthionine sulfoximine (BSO), a potent inhibitor of GCLC. BSO treatment led to a strong and significant decrease in endogenous glutathione levels (Fig. 6a). As above, a 1-h treatment with oxaliplatin alone was insufficient to kill all PMDOs. However, combination treatment of oxaliplatin with BSO resulted in complete eradication of two distinct PMDO cultures (Fig. 6b, c). BSO treatment caused a significant increase in Pt-DNA adduct formation by oxaliplatin which was blocked by the addition of exogenous glutathione (Fig. 6d). Combination treatment of oxaliplatin with BSO also led to a significant increase in DNA replication stress, as measured by the levels of pRPA (Fig. 6e).

Finally, we performed re-growth assays to assess the long-term regeneration potential of PMDOs following short-term treatment with oxaliplatin in the presence or absence of BSO (Fig. 6f). Two distinct PMDO cultures were treated with oxaliplatin alone for 1-h at clinically relevant concentrations. Following drug washout both cultures rapidly recovered and resumed growth within 3 weeks (Fig. 6g, h). BSO treatment alone had no effect on the recovery potential of PMDOs in the absence of oxaliplatin. However, a 1-h exposure to both oxaliplatin and BSO together significantly decreased the regenerative capacity of both PMDOs (Fig. 6g, h).

These results demonstrate the feasibility of killing PMDOs using short-term oxaliplatin treatment, but also that concomitant inhibition of glutathione synthesis is essential to achieve this.

## Discussion

In this report, we show that PM form a near-homogeneous entity of CMS4 tumours, a CRC subtype that is known to be relatively resistant to oxaliplatin [6–9]. PM-derived organoids were intrinsically resistant to clinically relevant concentrations of oxaliplatin. These findings may help explain, at least in part, why oxaliplatin-based HIPEC has failed to provide survival benefit in patients with operable PM in the Prodige7 study [10]. Importantly, this does not imply that adjuvant HIPEC should be abandoned as a potentially effective treatment strategy, as has been pointed out in various commentaries on the outcome of Prodige7 [34–36]. Indeed, the addition of HIPEC to surgery does provide a significant survival benefit in patients with PM derived from ovarian or gastric cancer [37–39].

Our study further demonstrates the feasibility of rationally identifying effective combination treatment strategies that allow eradication of PM-derived organoids with clinically achievable drug concentrations and short drug exposure times. Organoid technology currently provides the only pre-clinical platform on which clinical responses are faithfully reproduced [13, 16–19]. Moreover, colorectal PM-derived organoids have recently been used as a tool to guide therapy selection [40]. By combining molecular analysis of clinical PM tissue specimens and PM-derived organoids as a pre-clinical model system, we have identified glutathione synthesis as a major oxaliplatin resistance pathway. We propose that the addition of drugs that lower the reductive capacity of tumour cells to an oxaliplatin-based HIPEC regimen may increase treatment efficacy and improve local disease control in the peritoneum. Unfortunately, BSO is no longer being clinically applied due to its short in vivo half-life and its disappointing capacity to lower glutathione levels in tumour tissue [41]. An interesting alternative drug for targeting the GSH pathway is APR-246 [42], which is currently being tested in clinical trials. In addition, targeting the thioredoxin pathway may further synergise with GSH-targeting drugs in lowering the reductive capacity of tumour cells [43], in order to sensitise them to oxaliplatin and increase local treatment efficacy. To this end, Auranofin (an efficient inhibitor of thioredoxin reductase) is being evaluated in multiple clinical studies.

In addition to oxaliplatin, Mitomycin C (MMC) is frequently used in HIPEC. Interestingly, like oxaliplatin (this study), MMC can also be detoxified by glutahthione [44], suggesting that the combination treatment strategy identified in the present study may be beneficial for both oxaliplatin- and MMC-based HIPEC. Future studies should test the potentially synergistic effect of combining MMC with redox-targeting drugs on PMDOs.

The large-scale RNAseq analysis of PM from colorectal origin that is presented in this study provides many additional cues to the design of (combination) treatment strategies, not necessarily involving oxaliplatin. For instance, TGFβ signalling was identified as a major upregulated pathway in PM. High-level TGFβ signalling is characteristic of many stroma-rich tumour types, including CMS4 CRC [3] and is a major metastasis-promoting signalling pathway in late-stage CRC [45]. The clinical development of TGFβ signalling inhibitors is actively being pursued [46]. Within the peritoneum, TGF-β stimulates the generation of cancer-associated fibroblasts, which support the establishment of PM [47–50]. Inhibition of TGFβ signalling therefore represents an attractive alternative strategy for the treatment of CMS4 CRC, including PM. In addition, we have identified major differences in the tumour immune microenvironment between primary tumours and PM, with an enrichment of monocytes, macrophages and immature dendritic cells, and a strong reduction of T helper 17 cells in PM. These findings may form the basis for subsequent studies into the role of these distinct immune cell types in PM formation and resistance to therapy, and to what extent they may form targets for therapy in their own right. Macrophages in particular have been implicated in aggressive tumour behaviour including invasion, intravasation, tumour budding, and the generation of an immune-suppressed microenvironment [51]. Macrophages are enriched in CMS4 when compared to CMS1-3 in CRC [52] and macrophage-targeting therapies (reviewed in ref. [51]) may therefore have value in the treatment of PM.

In conclusion, through a combination of molecular analysis of clinical PM samples, and empirical studies using PMDOs, we have identified a major oxaliplatin resistance pathway in PM and provide an effective combination treatment strategy to overcome this resistance. The universally high expression of GCLC in all PMDOs suggests that lowering the reductive capacity of PM may be broadly applicable in patients with colorectal PM. Therapeutic approaches in which oxaliplatin is combined with redox-targeting drugs therefore deserve clinical evaluation in either PIPAC or HIPEC procedures. The PMDO drug screen results suggest that even with clinically relevant short-term exposure times, oxaliplatin treatment in combination with redox-targeting drugs may yield long-lasting anti-tumour effects.

## Supplementary information

Supplementary Figures 1-2

Supplemental Tables 1-3

checklist

## Data Availability

The datasets used and/or analysed during this study are available from the corresponding author on reasonable request.
