# Supplementary material for: Peritoneal metastases from colorectal cancer belong to Consensus Molecular Subtype 4 and are sensitised to oxaliplatin by inhibiting reducing capacity
Source: Br J Cancer. 2022 Feb 22;126(12):1824–33. doi: 10.1038/s41416-022-01742-5 (PMC9174226; doi:10.1038/s41416-022-01742-5)
Supplement: Supplementary file 1 — Supplementary Figures 1-2 [file 41416_2022_1742_MOESM1_ESM.docx]

**
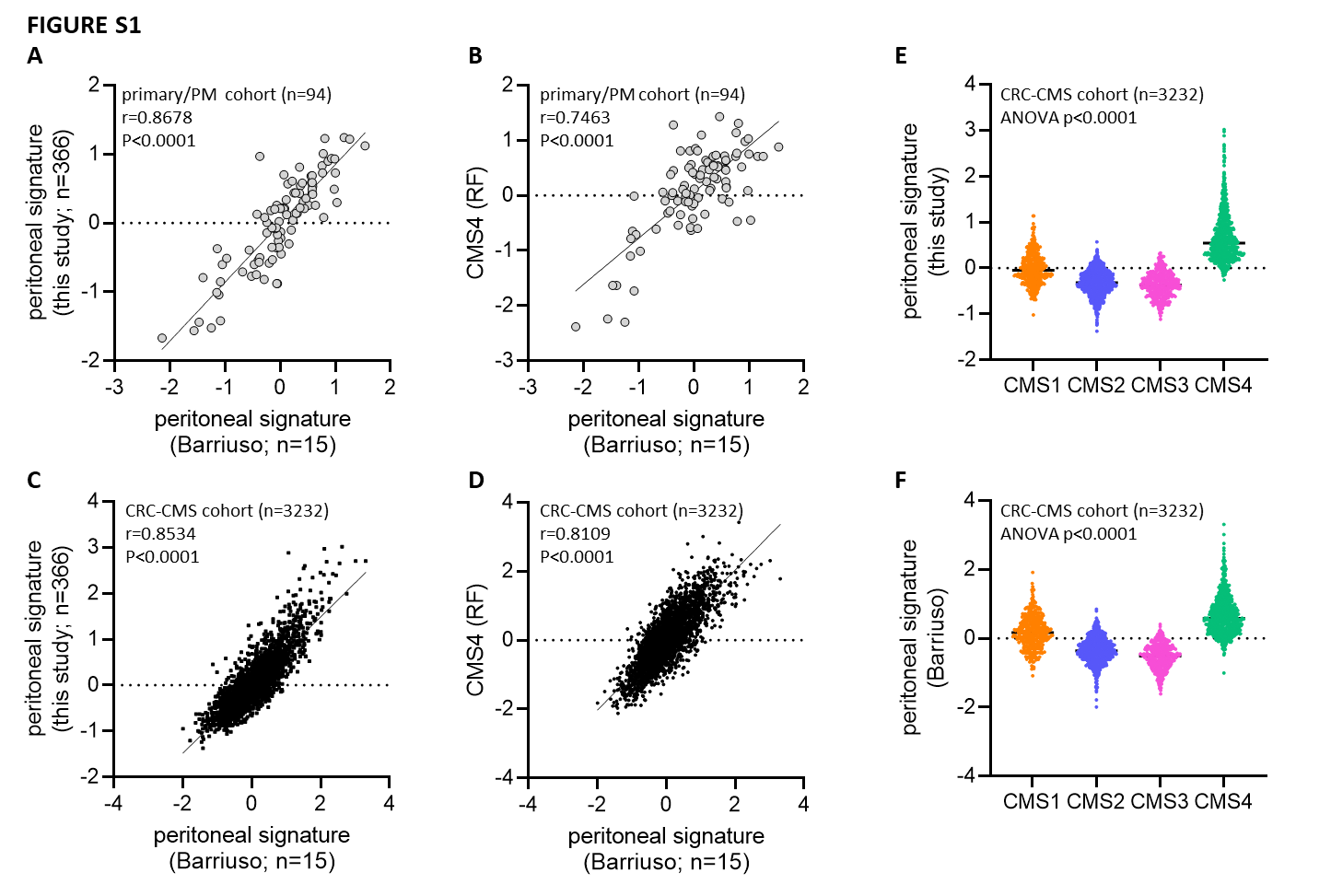
**

**Fig. S1 – Independently generated peritoneal signatures identify CMS4 CRC**

**a.** The RNAseq data generated in this study was used to compare expression of the 366-gene ‘peritoneal signature’ that was identified in this study in relation to expression of the peritoneal signature (15 genes) identified in the study by Barriuso et al. The scatter plot shows a highly significant correlation of both signatures. **b.** As in a., comparing expression of the Barriuso peritoneal signature with the CMS4-identfying genes from the original random forest classifier. The scatter plot shows a highly significant correlation of both gene sets. **c.** As in a. in the CMS cohort containing 3232 primary tumors. **d.** As in b, in the CMS cohort. **e.** Expression of the peritoneal signature identified in our study across CMS subtypes in the CMS cohort. **f.** Expression of the peritoneal signature identified by Barriuso et al across CMS subtypes in the CMS cohort.

**
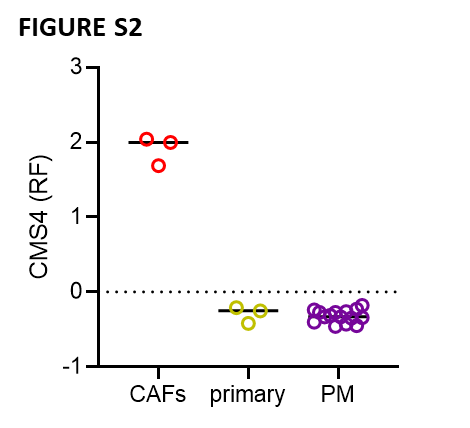
**

**Fig. S2 – Expression of CMS4 classifier genes in cancer-associated fibroblasts but not in organoids**

RNAseq analysis of cancer-associated fibroblasts (CAFs) and organoids was performed to assess expression of CMS4-identifying genes from the random forest classifier in CAFs (n=3) and in organoids derived from primary tumors (n=3) and PM (n=15). P value [primary vs. CAF] <0.0001. P value [PM vs. CAF] <0.0001. P value [primary vs. PM] not significant.
