# Supplementary material for: Peritoneal metastases from colorectal cancer belong to Consensus Molecular Subtype 4 and are sensitised to oxaliplatin by inhibiting reducing capacity
Source: Br J Cancer. 2022 Feb 22;126(12):1824–33. doi: 10.1038/s41416-022-01742-5 (PMC9174226; doi:10.1038/s41416-022-01742-5)
Supplement: Supplementary file 2 — Supplemental Tables 1-3 [file 41416_2022_1742_MOESM2_ESM.docx]

**Table S1.** Cohort of patients with peritoneal metastases and paired primary colorectal tumors.

| **location primary tumor** | **pT** | **pN** | **pM** | **mutational status tumor** | **#primary tumor regions** | **# peritoneal metastases** |
| --- | --- | --- | --- | --- | --- | --- |
|  |  |  |  |  |  |  |
| cecum | T4 | N2 | M1 | BRAF | 3 | 10 |
|  |  |  |  |  |  |  |
| cecum | T3 | N1 | M1 | NA | 3 | 2 |
|  |  |  |  |  |  |  |
| cecum | T3 | N2 | M1 | KRAS, PIK3CA | 2 | 12 |
|  |  |  |  |  |  |  |
| rectum | T3 | N1 | M1 | NA | 3 | 4 |
|  |  |  |  |  |  |  |
| sigmoid | T3 | N0 | M1 | KRAS, TP53 | 3 | 2 |
|  |  |  |  |  |  |  |
| cecum | T4 | N2 | M1 | BRAF, SMAD4, TP53 | 3 | 2 |
|  |  |  |  |  |  |  |
| transverse colon | T4 | N2 | M1 | KRAS, SMAD4, TP53 | 3 | 2 |
|  |  |  |  |  |  |  |
|  | T3 | N2 | M1 | ERBB2, TP53 | 2 | 1 |
|  |  |  |  |  |  |  |
| ascending colon | T4 | N2 | M1 | NRAS, TP53 | 4 | 4 |
|  |  |  |  |  |  |  |
| cecum | T3 | N2 | M1 | PIK3CA, TP53 | 2 | 9 |
|  |  |  |  |  |  |  |
| cecum | T4 | N2 | M1 | BRAF, MHL1 promotor hypermethylation | 3 | 7 |
|  |  |  |  |  |  |  |
| sigmoid | T4 | N1 | M1 | NA | 3 | 4 |

**Table S2.** Differentially expressed genes in primary CRC and paired peritoneal metastases (p<0.01 with FDR correction)

| **HIGHER in PM** | **p** | **fold difference** |  | **HIGHER in primary tumor** | **p** | **fold difference** |
| --- | --- | --- | --- | --- | --- | --- |
| FCN2 | 2,70E-03 | -7,17 |  | IL17F | 3,36E-03 | 21,05 |
| AC010969,1 | 3,17E-03 | -4,74 |  | IL17A | 6,90E-05 | 14,18 |
| CSN1S1 | 6,22E-03 | -4,39 |  | RPS2P2 | 6,12E-03 | 4,95 |
| FAM180B | 1,82E-03 | -4,36 |  | RP11-316M21,7 | 3,27E-03 | 4,41 |
| MT-TA | 2,02E-03 | -4,33 |  | RP6-159A1,3 | 8,81E-03 | 4,32 |
| SLC7A10 | 7,55E-05 | -3,81 |  | LINC01082 | 1,34E-03 | 4,31 |
| NT5C1A | 8,84E-03 | -3,63 |  | IL24 | 7,34E-05 | 4,24 |
| VTRNA2-1 | 7,91E-03 | -3,61 |  | C9ORF57 | 3,37E-03 | 3,77 |
| GOLGA8IP | 8,22E-03 | -3,54 |  | RP11-474D1,4 | 5,66E-04 | 3,45 |
| AL035610,1 | 3,61E-03 | -3,49 |  | RP11-392O1,4 | 5,93E-03 | 2,88 |
| CIDEA | 7,20E-05 | -3,47 |  | AC022182,1 | 1,00E-03 | 2,77 |
| MT-TQ | 2,14E-03 | -3,18 |  | RP5-1106E3,1 | 9,77E-03 | 2,69 |
| CPA1 | 1,39E-03 | -3,14 |  | KRTAP5-5 | 5,89E-03 | 2,44 |
| TUSC5 | 3,97E-05 | -3,02 |  | SLC25A47 | 6,03E-03 | 2,39 |
| SLC25A18 | 3,91E-03 | -2,90 |  | GFI1B | 3,97E-05 | 2,37 |
| ADIPOQ | 6,40E-06 | -2,82 |  | MMP3 | 5,88E-08 | 2,27 |
| C14ORF180 | 2,03E-03 | -2,81 |  | RP11-514D23,3 | 1,79E-04 | 2,26 |
| ACADL | 4,28E-05 | -2,77 |  | RP11-275I14,4 | 8,52E-04 | 2,26 |
| HEPACAM | 7,44E-04 | -2,68 |  | CTB-43E15,2 | 9,85E-03 | 2,22 |
| MRAP | 3,30E-04 | -2,65 |  | GRIA4 | 8,09E-03 | 2,20 |
| THRSP | 1,90E-04 | -2,65 |  | FAM150A | 6,80E-04 | 2,19 |
| RP11-667K14,4 | 3,52E-03 | -2,59 |  | RP11-645C24,4 | 3,94E-03 | 2,19 |
| CD300LG | 2,14E-04 | -2,55 |  | DUOXA2 | 5,65E-04 | 2,12 |
| MIRLET7D | 8,35E-03 | -2,54 |  | OR7E47P | 2,07E-04 | 2,12 |
| SGCG | 2,45E-04 | -2,40 |  | RP11-467L20,10 | 5,94E-03 | 2,08 |
| EPGN | 3,79E-03 | -2,39 |  | AC007099,1 | 9,17E-03 | 2,07 |
| RP3-483K16,4 | 8,51E-03 | -2,33 |  | AC007405,4 | 1,29E-03 | 2,06 |
| AC005550,4 | 3,28E-03 | -2,30 |  | RP11-503C24,6 | 1,70E-03 | 2,06 |
| MT-TP | 2,35E-03 | -2,29 |  | RP11-474D1,3 | 1,04E-03 | 2,02 |
| TCF23 | 2,65E-04 | -2,22 |  | SPINK4 | 9,91E-04 | 1,92 |
| KLF14 | 6,18E-03 | -2,21 |  | AKR1C4 | 5,65E-03 | 1,86 |
| GATA5 | 4,62E-03 | -2,18 |  | GATA1 | 9,88E-03 | 1,81 |
| MAG | 8,69E-03 | -2,14 |  | FABP2 | 8,41E-03 | 1,80 |
| ASTN1 | 1,61E-03 | -2,07 |  | VAC14-AS1 | 1,87E-04 | 1,78 |
| EGR4 | 1,96E-03 | -2,06 |  | FCAMR | 6,20E-03 | 1,71 |
| ASIP | 4,63E-03 | -2,05 |  | PLA2G4D | 2,80E-04 | 1,66 |
| FAT2 | 8,21E-03 | -2,04 |  | AC000068,9 | 3,38E-03 | 1,64 |
| TMEM132C | 5,29E-04 | -2,04 |  | RNF183 | 5,98E-05 | 1,59 |
| CPA4 | 5,84E-04 | -2,02 |  | TERT | 5,15E-03 | 1,59 |
| TRPM3 | 2,07E-03 | -2,01 |  | UCN2 | 6,42E-04 | 1,58 |
| RP11-893F2,6 | 9,22E-03 | -1,96 |  | RSPH14 | 2,15E-03 | 1,57 |
| BNC1 | 2,12E-03 | -1,94 |  | CCR3 | 3,24E-03 | 1,57 |
| CNTFR | 2,98E-04 | -1,94 |  | C4BPA | 3,22E-04 | 1,55 |
| TMEM151A | 3,10E-03 | -1,89 |  | OR2A7 | 7,04E-03 | 1,51 |
| KCNA1 | 8,43E-04 | -1,89 |  | TRPA1 | 4,80E-04 | 1,50 |
| LEP | 6,36E-04 | -1,89 |  | CXCL5 | 8,69E-03 | 1,50 |
| TNNT3 | 1,86E-03 | -1,87 |  | MS4A8 | 6,09E-03 | 1,49 |
| TPRG1-AS1 | 3,19E-03 | -1,87 |  | HOTTIP | 3,18E-03 | 1,44 |
| TCEAL5 | 7,91E-03 | -1,86 |  | MEP1A | 7,97E-03 | 1,44 |
| HP | 1,18E-03 | -1,85 |  | FENDRR | 2,40E-04 | 1,43 |
| VIT | 6,22E-03 | -1,83 |  | RP11-546J1,1 | 5,89E-03 | 1,42 |
| PLIN1 | 5,49E-05 | -1,81 |  | SYNPR-AS1 | 8,01E-03 | 1,41 |
| PRG4 | 7,45E-05 | -1,81 |  | CASP5 | 2,19E-03 | 1,39 |
| KRT5 | 1,88E-03 | -1,79 |  | PLEKHG7 | 3,17E-03 | 1,38 |
| HCAR1 | 2,14E-04 | -1,79 |  | CTD-2334D19,1 | 5,87E-03 | 1,37 |
| SIGLEC22P | 6,98E-03 | -1,79 |  | CTD-2227E11,1 | 1,59E-03 | 1,35 |
| PLCXD3 | 6,82E-03 | -1,79 |  | DUOX2 | 1,22E-03 | 1,35 |
| PRRT4 | 1,01E-03 | -1,77 |  | RP11-341G23,4 | 2,21E-03 | 1,31 |
| NAT8L | 3,82E-06 | -1,77 |  | RPS7P3 | 4,74E-03 | 1,31 |
| WT1-AS | 9,70E-04 | -1,76 |  | SNORD3A | 5,71E-03 | 1,30 |
| GREB1L | 1,09E-04 | -1,75 |  | COL28A1 | 6,31E-03 | 1,30 |
| ADAMTS9-AS1 | 3,09E-04 | -1,75 |  | HOXA11 | 3,76E-03 | 1,29 |
| SLITRK2 | 6,90E-04 | -1,75 |  | A1CF | 6,68E-03 | 1,29 |
| LINC01018 | 3,82E-03 | -1,73 |  | HOXA11-AS | 4,15E-03 | 1,28 |
| SCN4A | 1,59E-03 | -1,73 |  | RP11-328M4,2 | 4,16E-03 | 1,27 |
| SLITRK5 | 6,99E-03 | -1,73 |  | RP11-108L7,15 | 3,17E-03 | 1,26 |
| RP11-259P15,4 | 4,73E-03 | -1,73 |  | MRAP2 | 6,48E-03 | 1,26 |
| MS4A4E | 4,73E-03 | -1,72 |  | KCNJ11 | 6,65E-03 | 1,25 |
| LINC00989 | 7,78E-03 | -1,72 |  | AC007405,6 | 8,27E-03 | 1,25 |
| DNM1P47 | 8,35E-03 | -1,69 |  | AC021218,2 | 9,69E-03 | 1,25 |
| PI16 | 3,17E-03 | -1,69 |  | RSPH1 | 7,04E-03 | 1,24 |
| C6 | 3,98E-03 | -1,68 |  | LCN12 | 3,27E-03 | 1,24 |
| PPP1R1A | 1,35E-03 | -1,66 |  | AZGP1 | 3,73E-03 | 1,21 |
| PCDHA12 | 9,88E-03 | -1,66 |  | RP3-325F22,5 | 9,13E-03 | 1,21 |
| HPCAL4 | 7,68E-03 | -1,66 |  | AC004463,6 | 5,85E-03 | 1,21 |
| FABP4 | 1,27E-05 | -1,65 |  | RORC | 9,86E-03 | 1,17 |
| LINC00607 | 3,33E-04 | -1,64 |  | SAMD5 | 1,39E-03 | 1,16 |
| RP11-62F24,2 | 6,97E-03 | -1,63 |  | FOXF1 | 4,73E-03 | 1,15 |
| SUGT1P1 | 4,90E-03 | -1,63 |  | COL7A1 | 6,98E-04 | 1,15 |
| AL132709,5 | 7,09E-03 | -1,63 |  | RP4-758J18,13 | 3,12E-03 | 1,15 |
| CCDC39 | 8,84E-03 | -1,63 |  | IQCH | 6,68E-03 | 1,15 |
| COL25A1 | 3,28E-03 | -1,62 |  | JPH1 | 7,55E-03 | 1,14 |
| LBP | 8,36E-03 | -1,61 |  | CDCA7 | 7,54E-03 | 1,14 |
| KLB | 6,60E-03 | -1,60 |  | PMEL | 1,71E-03 | 1,14 |
| C5ORF49 | 6,82E-03 | -1,60 |  | SERPINA1 | 6,25E-03 | 1,11 |
| PCOLCE2 | 4,77E-06 | -1,59 |  | WNT5A | 2,47E-03 | 1,11 |
| RP11-472N13,3 | 3,11E-03 | -1,58 |  | RP11-152N13,5 | 6,05E-03 | 1,10 |
| CTD-2619J13,9 | 6,82E-03 | -1,58 |  | LNX1 | 7,33E-03 | 1,10 |
| NTNG1 | 8,21E-03 | -1,57 |  | RP11-295G20,2 | 9,30E-03 | 1,10 |
| ANKRD45 | 5,20E-03 | -1,57 |  | NUDT7 | 4,57E-03 | 1,09 |
| KRBOX1 | 6,39E-04 | -1,56 |  | C1ORF226 | 6,56E-03 | 1,09 |
| HAS1 | 6,17E-04 | -1,56 |  | SNHG10 | 2,62E-03 | 1,09 |
| RP11-733O18,1 | 7,54E-03 | -1,55 |  | EPHX2 | 8,43E-03 | 1,09 |
| AP000476,1 | 2,25E-03 | -1,55 |  | NQO1 | 8,85E-03 | 1,09 |
| MT-TT | 4,37E-04 | -1,54 |  | RP11-18I14,10 | 4,92E-03 | 1,08 |
| SIX2 | 5,44E-03 | -1,53 |  | METTL12 | 4,64E-03 | 1,07 |
| PLIN4 | 2,70E-04 | -1,53 |  | PINX1 | 1,34E-03 | 1,07 |
| CCIN | 5,39E-03 | -1,53 |  | HDHD3 | 9,38E-03 | 1,07 |
| TSKS | 9,35E-03 | -1,53 |  | UBE3D | 5,81E-03 | 1,07 |
| RP11-413E6,1 | 1,35E-03 | -1,52 |  | HSD17B7 | 6,84E-03 | 1,07 |
| MED12L | 6,07E-05 | -1,50 |  | IMMP2L | 2,99E-03 | 1,07 |
| CRHBP | 9,32E-03 | -1,50 |  | SORD | 5,98E-03 | 1,07 |
| MYOZ1 | 3,90E-03 | -1,50 |  | ZNF511 | 1,31E-03 | 1,06 |
| MUM1L1 | 3,21E-03 | -1,49 |  | TIMM8A | 7,88E-03 | 1,06 |
| CNKSR2 | 5,21E-03 | -1,49 |  | CHEK2 | 2,97E-03 | 1,06 |
| GALNT13 | 3,74E-03 | -1,49 |  | DECR2 | 8,82E-03 | 1,06 |
| ZNF676 | 5,25E-03 | -1,49 |  | PPAT | 1,95E-03 | 1,05 |
| ADD2 | 4,12E-05 | -1,48 |  | OARD1 | 2,77E-03 | 1,05 |
| GRIN2A | 5,81E-03 | -1,48 |  | CDC25B | 2,96E-03 | 1,05 |
| RP11-834C11,7 | 1,98E-03 | -1,47 |  | PAICS | 1,39E-03 | 1,05 |
| FOSB | 3,55E-08 | -1,46 |  | UPF3B | 1,39E-03 | 1,05 |
| SFRP1 | 2,36E-04 | -1,46 |  | LARS2 | 5,82E-03 | 1,05 |
| CIDEC | 8,46E-04 | -1,46 |  | COA6 | 3,61E-03 | 1,04 |
| CADM3 | 2,34E-04 | -1,46 |  | POLA1 | 9,09E-03 | 1,04 |
| VSTM2L | 2,98E-04 | -1,46 |  | RMND1 | 9,77E-03 | 1,04 |
| WT1 | 2,11E-03 | -1,44 |  | CISD1 | 9,44E-03 | 1,04 |
| LINC00565 | 8,23E-03 | -1,44 |  | AFMID | 7,42E-03 | 1,04 |
| KCNAB1 | 4,80E-04 | -1,44 |  | H1F0 | 6,16E-03 | 1,04 |
| ADH1B | 1,96E-03 | -1,43 |  | TECR | 8,91E-03 | 1,04 |
| PRSS35 | 1,42E-03 | -1,43 |  | PA2G4 | 2,44E-03 | 1,04 |
| ACSM5 | 1,34E-03 | -1,42 |  | PATZ1 | 1,96E-03 | 1,04 |
| FMO1 | 2,71E-03 | -1,42 |  | FH | 6,91E-03 | 1,04 |
| FMO2 | 8,51E-04 | -1,40 |  | NAT10 | 7,35E-03 | 1,04 |
| EXTL1 | 9,45E-03 | -1,40 |  | COX15 | 8,11E-04 | 1,04 |
| NGFR | 4,83E-04 | -1,38 |  | IFT172 | 5,88E-03 | 1,03 |
| SLITRK4 | 2,01E-03 | -1,38 |  | IMPDH2 | 6,35E-03 | 1,03 |
| ADRA1B | 1,07E-03 | -1,37 |  | NOLC1 | 9,15E-03 | 1,03 |
| ASPA | 1,69E-03 | -1,37 |  | LAS1L | 5,13E-03 | 1,03 |
| GALNT16 | 5,97E-03 | -1,36 |  | SNRPD3 | 3,11E-03 | 1,03 |
| HRASLS5 | 6,06E-03 | -1,36 |  | PQBP1 | 4,15E-03 | 1,03 |
| AC090587,4 | 2,68E-03 | -1,35 |  | CCT3 | 7,56E-03 | 1,03 |
| RGS4 | 1,31E-05 | -1,33 |  | RSL1D1 | 7,07E-03 | 1,03 |
| ADGRG2 | 9,45E-03 | -1,33 |  | ST13 | 8,55E-03 | 1,02 |
| ABCA8 | 3,74E-04 | -1,33 |  | FUBP1 | 4,14E-03 | 1,02 |
| AGTR1 | 5,96E-03 | -1,32 |  | DAP3 | 8,49E-03 | 1,02 |
| DIRAS1 | 2,98E-03 | -1,32 |  | COPZ1 | 4,39E-03 | 1,02 |
| CTD-2162K18,5 | 9,32E-03 | -1,32 |  | RBMX | 3,87E-03 | 1,02 |
| EBF2 | 1,84E-03 | -1,32 |  | HMGN2P21 | 2,52E-03 | 0,00 |
| NGF | 2,25E-03 | -1,32 |  |  |  |  |
| CLDN11 | 9,66E-04 | -1,31 |  |  |  |  |
| BCHE | 1,42E-03 | -1,31 |  |  |  |  |
| PKNOX2 | 9,78E-03 | -1,30 |  |  |  |  |
| C7 | 2,42E-03 | -1,30 |  |  |  |  |
| OSR1 | 2,26E-03 | -1,30 |  |  |  |  |
| RYR1 | 1,72E-04 | -1,29 |  |  |  |  |
| IL10 | 4,63E-03 | -1,29 |  |  |  |  |
| LRRN1 | 9,05E-03 | -1,29 |  |  |  |  |
| PAK3 | 1,96E-03 | -1,29 |  |  |  |  |
| PLAC9 | 5,21E-04 | -1,29 |  |  |  |  |
| RBP7 | 3,13E-04 | -1,29 |  |  |  |  |
| KLHL4 | 1,60E-03 | -1,28 |  |  |  |  |
| GPIHBP1 | 4,40E-03 | -1,28 |  |  |  |  |
| CRABP2 | 2,50E-03 | -1,27 |  |  |  |  |
| FOXC2 | 9,00E-03 | -1,26 |  |  |  |  |
| ABCA9 | 2,95E-03 | -1,26 |  |  |  |  |
| CDH23 | 8,25E-04 | -1,25 |  |  |  |  |
| NAV3 | 1,05E-03 | -1,25 |  |  |  |  |
| CORO2B | 7,50E-04 | -1,25 |  |  |  |  |
| KCNJ5 | 6,62E-03 | -1,25 |  |  |  |  |
| UCHL1 | 2,12E-04 | -1,24 |  |  |  |  |
| SNX32 | 7,98E-03 | -1,24 |  |  |  |  |
| NR4A3 | 4,91E-03 | -1,24 |  |  |  |  |
| ADAMTS15 | 6,29E-04 | -1,23 |  |  |  |  |
| CD36 | 7,31E-05 | -1,23 |  |  |  |  |
| NTRK2 | 3,66E-03 | -1,23 |  |  |  |  |
| MEOX2 | 4,44E-03 | -1,23 |  |  |  |  |
| PCDHGA12 | 4,03E-03 | -1,23 |  |  |  |  |
| LRRC4 | 8,80E-03 | -1,23 |  |  |  |  |
| ARL4D | 2,59E-04 | -1,22 |  |  |  |  |
| HSPB6 | 7,59E-03 | -1,22 |  |  |  |  |
| KCNT2 | 9,51E-03 | -1,22 |  |  |  |  |
| CYP1B1 | 1,84E-03 | -1,22 |  |  |  |  |
| CLSTN2 | 4,95E-03 | -1,22 |  |  |  |  |
| PIEZO2 | 1,39E-04 | -1,21 |  |  |  |  |
| ZNF229 | 5,66E-03 | -1,21 |  |  |  |  |
| ZFPM2 | 6,81E-04 | -1,20 |  |  |  |  |
| RP11-572C15,6 | 2,94E-03 | -1,20 |  |  |  |  |
| IGF1 | 3,20E-03 | -1,20 |  |  |  |  |
| EGR3 | 3,95E-03 | -1,20 |  |  |  |  |
| ATP8B3 | 4,12E-03 | -1,20 |  |  |  |  |
| EGR2 | 1,60E-03 | -1,19 |  |  |  |  |
| SRPX | 1,59E-03 | -1,19 |  |  |  |  |
| C19ORF26 | 6,45E-03 | -1,19 |  |  |  |  |
| TDRD9 | 8,85E-03 | -1,19 |  |  |  |  |
| LIPE | 2,59E-03 | -1,19 |  |  |  |  |
| OPRL1 | 3,20E-03 | -1,19 |  |  |  |  |
| TPRG1 | 1,85E-03 | -1,19 |  |  |  |  |
| ABCA6 | 5,42E-03 | -1,19 |  |  |  |  |
| PDE1B | 2,29E-03 | -1,18 |  |  |  |  |
| C17ORF107 | 4,65E-03 | -1,18 |  |  |  |  |
| MFAP5 | 2,78E-03 | -1,18 |  |  |  |  |
| ADCY1 | 6,87E-04 | -1,18 |  |  |  |  |
| MEDAG | 1,83E-03 | -1,18 |  |  |  |  |
| LINC00341 | 2,68E-03 | -1,18 |  |  |  |  |
| PDK4 | 2,42E-04 | -1,18 |  |  |  |  |
| CFD | 1,23E-03 | -1,18 |  |  |  |  |
| PTGIS | 3,50E-03 | -1,18 |  |  |  |  |
| FAM69B | 1,71E-03 | -1,17 |  |  |  |  |
| ANKRD29 | 6,98E-03 | -1,17 |  |  |  |  |
| SLIT2 | 3,38E-03 | -1,17 |  |  |  |  |
| GHR | 2,58E-03 | -1,17 |  |  |  |  |
| TGFB3 | 5,14E-03 | -1,17 |  |  |  |  |
| CXORF21 | 3,36E-03 | -1,16 |  |  |  |  |
| FBXL2 | 3,21E-03 | -1,16 |  |  |  |  |
| ZNF287 | 1,36E-03 | -1,16 |  |  |  |  |
| RP5-1042I8,7 | 4,98E-03 | -1,16 |  |  |  |  |
| GUCY1A2 | 4,76E-04 | -1,16 |  |  |  |  |
| NPR1 | 2,58E-03 | -1,15 |  |  |  |  |
| NPTXR | 1,90E-03 | -1,15 |  |  |  |  |
| ANKRD6 | 1,95E-03 | -1,15 |  |  |  |  |
| ZNF829 | 5,23E-03 | -1,15 |  |  |  |  |
| CDON | 3,51E-03 | -1,15 |  |  |  |  |
| LPL | 1,41E-03 | -1,15 |  |  |  |  |
| PCDHGB7 | 8,51E-03 | -1,14 |  |  |  |  |
| NFATC1 | 4,00E-03 | -1,14 |  |  |  |  |
| ID4 | 7,07E-04 | -1,14 |  |  |  |  |
| LARP6 | 1,64E-03 | -1,14 |  |  |  |  |
| GPX3 | 1,59E-03 | -1,14 |  |  |  |  |
| NR4A2 | 5,35E-03 | -1,14 |  |  |  |  |
| RP11-420L9,5 | 4,54E-03 | -1,14 |  |  |  |  |
| CCDC80 | 4,90E-03 | -1,14 |  |  |  |  |
| EGR1 | 1,45E-05 | -1,14 |  |  |  |  |
| CTC-205M6,5 | 4,75E-03 | -1,14 |  |  |  |  |
| HPGDS | 9,36E-03 | -1,14 |  |  |  |  |
| LINC00936 | 5,23E-03 | -1,14 |  |  |  |  |
| DENND2C | 5,29E-03 | -1,13 |  |  |  |  |
| ZFHX2 | 2,46E-03 | -1,13 |  |  |  |  |
| KLF2 | 3,27E-04 | -1,13 |  |  |  |  |
| TIAM1 | 1,97E-03 | -1,13 |  |  |  |  |
| FOS | 6,22E-05 | -1,13 |  |  |  |  |
| NINL | 3,85E-03 | -1,13 |  |  |  |  |
| COPZ2 | 8,85E-03 | -1,13 |  |  |  |  |
| MMP19 | 9,22E-03 | -1,13 |  |  |  |  |
| MIR22HG | 6,66E-05 | -1,13 |  |  |  |  |
| C1ORF162 | 8,83E-03 | -1,13 |  |  |  |  |
| ROR1 | 7,15E-03 | -1,13 |  |  |  |  |
| DUSP1 | 2,45E-04 | -1,13 |  |  |  |  |
| ZNF423 | 4,97E-03 | -1,12 |  |  |  |  |
| PALMD | 5,20E-03 | -1,12 |  |  |  |  |
| ZNF331 | 4,18E-04 | -1,12 |  |  |  |  |
| GPR183 | 3,59E-03 | -1,12 |  |  |  |  |
| MAP1B | 2,83E-04 | -1,12 |  |  |  |  |
| ZFP82 | 1,33E-03 | -1,12 |  |  |  |  |
| HECTD2 | 7,08E-03 | -1,12 |  |  |  |  |
| ZDBF2 | 2,57E-03 | -1,12 |  |  |  |  |
| RGCC | 9,24E-04 | -1,12 |  |  |  |  |
| LY86 | 8,35E-03 | -1,12 |  |  |  |  |
| ZNF549 | 6,24E-03 | -1,12 |  |  |  |  |
| NAP1L5 | 8,08E-03 | -1,12 |  |  |  |  |
| NR4A1 | 7,08E-03 | -1,12 |  |  |  |  |
| ECM2 | 9,49E-03 | -1,12 |  |  |  |  |
| ZNF354C | 9,46E-03 | -1,11 |  |  |  |  |
| GPSM1 | 5,42E-03 | -1,11 |  |  |  |  |
| ABLIM3 | 6,92E-03 | -1,11 |  |  |  |  |
| RP11-469M7,1 | 2,70E-03 | -1,11 |  |  |  |  |
| CYR61 | 4,24E-03 | -1,11 |  |  |  |  |
| SLC46A1 | 7,26E-05 | -1,11 |  |  |  |  |
| SGK1 | 5,14E-04 | -1,11 |  |  |  |  |
| TMEM86A | 2,11E-03 | -1,11 |  |  |  |  |
| VLDLR | 3,52E-03 | -1,11 |  |  |  |  |
| DCBLD2 | 2,00E-03 | -1,10 |  |  |  |  |
| PDLIM3 | 5,87E-03 | -1,10 |  |  |  |  |
| C20ORF194 | 2,79E-03 | -1,10 |  |  |  |  |
| ERG | 7,54E-03 | -1,10 |  |  |  |  |
| OLFML2B | 9,37E-03 | -1,10 |  |  |  |  |
| CD83 | 5,79E-03 | -1,10 |  |  |  |  |
| TUBB2A | 4,00E-03 | -1,10 |  |  |  |  |
| MDFI | 7,60E-03 | -1,09 |  |  |  |  |
| TUBB6 | 1,36E-03 | -1,09 |  |  |  |  |
| RASSF2 | 3,96E-03 | -1,09 |  |  |  |  |
| PHLDB2 | 7,84E-03 | -1,09 |  |  |  |  |
| TUBA1A | 5,00E-04 | -1,09 |  |  |  |  |
| MYO5A | 2,79E-03 | -1,09 |  |  |  |  |
| NAT14 | 5,43E-03 | -1,09 |  |  |  |  |
| GADD45B | 2,42E-03 | -1,09 |  |  |  |  |
| ADAP2 | 8,82E-03 | -1,09 |  |  |  |  |
| CSRNP1 | 3,82E-03 | -1,09 |  |  |  |  |
| VASH1 | 9,44E-03 | -1,08 |  |  |  |  |
| AC096772,6 | 4,73E-04 | -1,08 |  |  |  |  |
| CTGF | 7,35E-03 | -1,08 |  |  |  |  |
| KIF3C | 8,89E-03 | -1,08 |  |  |  |  |
| GPC1 | 2,96E-03 | -1,08 |  |  |  |  |
| PXDC1 | 4,15E-03 | -1,08 |  |  |  |  |
| SYNPO | 1,95E-03 | -1,08 |  |  |  |  |
| NAV1 | 8,75E-03 | -1,08 |  |  |  |  |
| JUNB | 7,33E-05 | -1,07 |  |  |  |  |
| PIM1 | 2,70E-03 | -1,07 |  |  |  |  |
| ANXA1 | 4,10E-03 | -1,07 |  |  |  |  |
| NATD1 | 7,53E-03 | -1,07 |  |  |  |  |
| RASGEF1B | 9,16E-03 | -1,07 |  |  |  |  |
| ULK2 | 8,23E-03 | -1,07 |  |  |  |  |
| KATNAL1 | 2,58E-03 | -1,07 |  |  |  |  |
| ID3 | 7,25E-03 | -1,07 |  |  |  |  |
| DOCK4 | 4,13E-03 | -1,07 |  |  |  |  |
| ZNF684 | 7,56E-03 | -1,07 |  |  |  |  |
| EMP1 | 4,80E-04 | -1,07 |  |  |  |  |
| JDP2 | 8,83E-03 | -1,07 |  |  |  |  |
| WBP5 | 9,99E-03 | -1,06 |  |  |  |  |
| MYADM | 8,72E-03 | -1,06 |  |  |  |  |
| AMOTL2 | 9,23E-05 | -1,06 |  |  |  |  |
| FOXO1 | 3,38E-03 | -1,06 |  |  |  |  |
| GABARAP | 4,63E-03 | -1,06 |  |  |  |  |
| TIPARP | 3,02E-03 | -1,06 |  |  |  |  |
| RHOB | 5,86E-03 | -1,06 |  |  |  |  |
| IER2 | 6,38E-04 | -1,06 |  |  |  |  |
| VIM | 8,71E-03 | -1,06 |  |  |  |  |
| SNX21 | 1,97E-03 | -1,06 |  |  |  |  |
| KDM6B | 8,36E-03 | -1,06 |  |  |  |  |
| LRP1 | 4,74E-03 | -1,05 |  |  |  |  |
| MT-ND5 | 3,45E-03 | -1,05 |  |  |  |  |
| KLF10 | 1,98E-03 | -1,05 |  |  |  |  |
| CHFR | 7,00E-03 | -1,05 |  |  |  |  |
| PHTF2 | 6,26E-03 | -1,05 |  |  |  |  |
| STAT5A | 4,14E-03 | -1,05 |  |  |  |  |
| CTNS | 4,25E-03 | -1,05 |  |  |  |  |
| WDR37 | 3,45E-03 | -1,05 |  |  |  |  |
| GSN | 9,31E-03 | -1,05 |  |  |  |  |
| KLF6 | 1,88E-03 | -1,05 |  |  |  |  |
| LATS2 | 5,99E-03 | -1,05 |  |  |  |  |
| MARCH2 | 8,75E-03 | -1,05 |  |  |  |  |
| DAB2 | 3,46E-03 | -1,05 |  |  |  |  |
| MAST4 | 7,64E-03 | -1,05 |  |  |  |  |
| CYTH3 | 7,06E-03 | -1,05 |  |  |  |  |
| ABL2 | 9,14E-03 | -1,05 |  |  |  |  |
| ITSN1 | 1,71E-03 | -1,05 |  |  |  |  |
| ZNF17 | 9,50E-03 | -1,05 |  |  |  |  |
| ZNF516 | 2,57E-03 | -1,05 |  |  |  |  |
| MEGF8 | 3,42E-03 | -1,05 |  |  |  |  |
| TOM1L2 | 5,44E-03 | -1,05 |  |  |  |  |
| ARAP1 | 3,17E-03 | -1,04 |  |  |  |  |
| RAP2B | 4,00E-03 | -1,04 |  |  |  |  |
| NDEL1 | 1,37E-03 | -1,04 |  |  |  |  |
| HGSNAT | 8,18E-04 | -1,04 |  |  |  |  |
| ZNF18 | 5,82E-03 | -1,04 |  |  |  |  |
| PQLC1 | 1,95E-03 | -1,04 |  |  |  |  |
| TSC22D2 | 6,33E-04 | -1,04 |  |  |  |  |
| KLF11 | 8,89E-03 | -1,04 |  |  |  |  |
| MAP2K4 | 5,93E-03 | -1,04 |  |  |  |  |
| TRAF3 | 2,36E-03 | -1,04 |  |  |  |  |
| MEF2A | 3,51E-03 | -1,04 |  |  |  |  |
| ZBTB22 | 6,42E-03 | -1,04 |  |  |  |  |
| NDST1 | 9,89E-03 | -1,04 |  |  |  |  |
| CHMP1B | 3,76E-03 | -1,04 |  |  |  |  |
| IFT20 | 3,37E-03 | -1,04 |  |  |  |  |
| ADAM17 | 1,34E-03 | -1,04 |  |  |  |  |
| ZNF329 | 4,17E-03 | -1,04 |  |  |  |  |
| TNFAIP1 | 5,38E-03 | -1,04 |  |  |  |  |
| MPRIP | 9,33E-03 | -1,03 |  |  |  |  |
| KCTD21 | 9,02E-03 | -1,03 |  |  |  |  |
| FRS2 | 2,53E-03 | -1,03 |  |  |  |  |
| DERL2 | 4,96E-03 | -1,03 |  |  |  |  |
| PRMT2 | 3,99E-03 | -1,03 |  |  |  |  |
| OPA3 | 3,74E-03 | -1,03 |  |  |  |  |
| SPAG7 | 2,12E-03 | -1,03 |  |  |  |  |
| CSRNP2 | 5,27E-03 | -1,03 |  |  |  |  |
| CTDP1 | 3,04E-03 | -1,03 |  |  |  |  |
| AKAP10 | 9,39E-03 | -1,03 |  |  |  |  |
| LRCH3 | 3,16E-03 | -1,03 |  |  |  |  |
| ANKFY1 | 5,89E-03 | -1,03 |  |  |  |  |
| TMEM43 | 2,46E-03 | -1,03 |  |  |  |  |
| BICD2 | 9,52E-03 | -1,03 |  |  |  |  |
| MTMR14 | 3,77E-03 | -1,03 |  |  |  |  |
| PAFAH1B1 | 2,73E-03 | -1,03 |  |  |  |  |
| CCDC127 | 7,05E-03 | -1,03 |  |  |  |  |
| WDR41 | 7,16E-03 | -1,03 |  |  |  |  |
| DVL3 | 5,91E-03 | -1,03 |  |  |  |  |

**Table S3.** Transcription factor binding site enrichment (2X2 contingency table analysis with continuity correction)

| **TF** | **# target genes** | **# in set** | **p** | **minus LOG10 P** |
| --- | --- | --- | --- | --- |
| ZEB1 | 2519 | 89 | 8,50E-06 | 5,1 |
| P300 | 6168 | 179 | 7,80E-05 | 4,1 |
| ZBTB3 | 4260 | 130 | 1,60E-04 | 3,8 |
| SF1 | 5174 | 152 | 2,40E-04 | 3,6 |
| GLIS1 | 3552 | 110 | 3,80E-04 | 3,4 |
| CREL | 3676 | 113 | 4,10E-04 | 3,4 |
| IPF1 | 1274 | 47 | 7,10E-04 | 3,1 |
| IPF1 | 1275 | 47 | 7,20E-04 | 3,1 |
| MEF2 | 1435 | 51 | 1,10E-03 | 3,0 |
| CEBP | 2354 | 76 | 1,30E-03 | 2,9 |
